# A Simple in Syringe Low Density Solvent-Dispersive Liquid Liquid Microextraction for Enrichment of Some Metal Ions Prior to Their Determination by High Performance Liquid Chromatography in Food Samples

**DOI:** 10.3390/molecules25030552

**Published:** 2020-01-28

**Authors:** Melasinee Laosuwan, Siriboon Mukdasai, Supalax Srijaranai

**Affiliations:** Materials Chemistry Research Center, Department of Chemistry and Center of Excellence for Innovation in Chemistry, Faculty of Science, Khon Kaen University, Khon Kaen 40002, Thailand; melasineemela@gmail.com (M.L.); sirimuk@kku.ac.th (S.M.)

**Keywords:** liquid phase microextraction, in-syringe microextraction, metal complex, pyrrolidine dithiocarbamate, simultaneous analysis

## Abstract

A simple and highly sensitive method is developed for the simultaneous determination of Ni^2+^, Cr_2_O_7_^2−^, Co^2+^, and Hg^2+^ by using in syringe low density solvent-dispersive liquid liquid microextraction (ISLD-DLLME), followed by high performance liquid chromatography with a UV detector. The four metal ions were derivatized with pyrrolidine dithiocarbamate (PDC) based on complexation before their enrichment by ISLD-DLLME in which 1-octanol and methanol were used as the extraction solvent and the dispersive solvent, respectively. The extraction was performed in a commercially available syringe under vortex agitation. Phase separation was achieved without centrifugation, and the extraction phase was easily collected by moving the syringe plunger. Parameters affecting the extraction efficiency were studied and optimized. Under the optimum conditions, the four metal-PDC complexes were detected within 18 min, and ISLD-DLLME could increase the detection sensitivity in the range of 64–230 times compared to the direct HPLC analysis. The obtained limits of detection (LODs) were found to be in the range of 0.011–2.0 µg L^−1^. The applicability of the method is demonstrated for freshwater fish, shrimp, and shellfish samples. In addition, the results are in good agreement with those obtained by inductively-coupled plasma-optical emission spectrometry (ICP-OES).

## 1. Introduction

The determination of metal ions is still an importance issue in many fields such as the environment and food sectors. [1]. The instrumental methods that are widely used for the determination of metal ions are based on atomic spectrophotometry like as atomic absorption spectrometry (AAS) [2,3], inductively-coupled plasma-optical emission spectrometry (ICP-OES) [4], and inductively-coupled plasma-mass spectrometry (ICP-MS) [5]. Recently, optical sensors have been employed for the detection of metal ions [6,7]. Though these methods are sensitive, highly precise and accurate, they are recognized as a “single-element” analytical technique. Multi-elemental analysis can be achieved with either ICP-OES or ICP-MS, but the instrument and operating costs are expensive. Another instrumental technique for the simultaneous analysis of metals is high performance liquid chromatography (HPLC). Various modes of HPLC have been successfully employed for the simultaneous analysis of metal ions such as ion exchange and the reversed phase. In addition, a number of hyphenated techniques for the HPLC analysis of metal ions, such as ion chromatography coupled with hydride generation–inductively-coupled plasma atomic emission spectrometry [8], ion chromatography [9] and high performance liquid chromatography coupled to inductively-coupled plasma mass spectrometry [10] have been reported. Most of chromatographic analysis of metal ions is performed after their pre-derivatization by complex formation with appropriate chelating agents. The complexation of metal ions provides not only selectivity that arises from the behavior of individual complexes but also sensitivity for absorption detection from the high absorption coefficient of metal complexes. 

The metal ions of interest in this study are Ni^2+^, Cr_2_O_7_^2−^, Co^2+^, and Hg^2+^. Ni^2+^ and Co^2+^, at trace concentrations, are essential for the human diet in order to maintain normal physiological functions [11]. At high concentrations, Ni^2+^ is highly toxic to the cardiovascular system and irritates the skin [12], while large amount of Co^2+^ affects breathing, asthma, pneumonia, wheezing, and skin rashes [13]. The level of toxicity of metals is dependent on their form (e.g., inorganic and organic) and oxidation states. For instance, Cr^6+^ is suspected to be a carcinogen and mutagenic compound [14], while Cr^3+^ is an essential trace element [15]. Inorganic mercury (Hg^2+^) is harmful to the tissue systems of humans [15]. These metal ions have been employed in various manufacturing processes such as those for paper, paint, plastic, electrochemical products, and pharmaceuticals, so they can contaminate the environment and the food chain [1]. To ensure food safety, regulations have been established by various authorized organizations. The maximum residue limit (MRL) of chromium in fish and shellfish has been regulated to be 2.0 mg kg^−1^ by United States Department of Agriculture (USDA) Foreign Agricultural Service [16]. The recommended MRL for Hg^2+^ by the European Union (EU) is in the range of 0.5–1.0 mg kg^−1^ fresh weight for fish muscle and other fishery products [17], while the dietary reference intake (DRI) of Ni^2+^ is regulated as less than 0.2 mg per day in food by the World Health Organization (WHO) [18]. Furthermore, the DRI allowance of Co^2+^ from food is estimated to be 5–40 µg per day by the Environmental Protection Agency (EPA) [19].

The determination of metal ions at trace levels can usually be achieved by an additional step that is known as the preconcentration method before the instrumental analysis. Various preconcentration techniques have been developed for the analysis of metal ions in order to increase sensitivity of the detection. Nowadays, the miniaturized approach of a classical liquid–liquid extraction (LLE), namely liquid phase microextraction (LPME), has gained extensive attention. Among various techniques of LPME, dispersive liquid–liquid microextraction (DLLME) has been accepted as the most popular technique because of its simplicity and rapidity. DLLME is composed of ternary solvents, including an aqueous solution containing the target analytes being extracted, an organic solvent as the extraction solvent, and a water-miscible organic solvent as the dispersive solvent [20,21]. The extraction occurs immediately after a rapid injection of a mixture of extraction and dispersive solvents into the aqueous phase, after which a cloudy phase is usually observed. Afterward, phase separation is then achieved by centrifugation, and the enriched analytes that are usually present in the sedimented phase are subsequently determined by appropriate techniques. There are a numbers of research works on DLLME for the determination of metal ions [22,23,24,25].

Likewise, for LLE, the extraction solvent is a key parameter for DLLME. In the first period of DLLME, solvents having higher densities than water, such as chlorinated solvents including chlorobenzene, carbon tetrachloride, and tetrachloroethylene, are used as the extraction solvents. However, these solvents are toxic [26]. Recently, to fulfill the green analytical concept, the solvents with lower densities than water, which are considered to be less toxic than those with higher densities, are used as the extraction solvent. DLLME, which employs a low-density extraction solvent, still has a problem in the collection of the extraction phase that presents as a floating drop at the top of the aqueous phase after centrifugation [26,27,28]. To overcome this problem, specially designed extraction vessels are used. Most of the specifically-designed extraction devices are either long-neck or narrow-neck vessels [20,29,30]. Thus far, the simplest reported extraction devices are syringes and syringes with pipet tip on top [31,32,33,34].

The aim of this study was to develop a simple and highly sensitive method for the simultaneous determination of Ni^2+^, Cr_2_O_7_^2−^, Co^2+^, and Hg^2+^ by HPLC. The method consists of the pre-derivatization of metal ions by complex formation with pyrrolidine dithiocarbamate (PDC), the preconcentration of the metal–PDC complexes by in syringe low density solvent-dispersive liquidliquid microextraction (ISLD-DLLME), and, finally, the determination of the enriched metal–PDC complexes by HPLC equipped with UV detector. The proposed method (ISLD-DLLME) followed by HPLC was then applied for the analysis of the metal ions in freshwater fish, shrimp and shellfish samples. In addition, ICP-OES was employed for comparison.

## 2. Results and Discussion 

PDC was chosen as the chelating agent to form complexes with the studied metal ions because of its high complexation capability with a number of metal ions and the fact that it provides high molar absorptivity complexes [35,36]. There are a number of reports on the determination of metal ions based on PDC-complexes by using various instrumental methods such as UV-Vis spectrophotometry [37], flame atomic absorption spectrometry (FAAS) [2], electrothermal atomic absorption spectrometry (ETAAS) [3], ICP-OES [4], ICP-MS [5] and HPLC [38,39]. In addition, PDC has been used to lower analytical limits of FAAS [40].

Generally, the anion of pyrrolidine dithiocarbamate forms neutral complexes with most metal ions, and it has been documented that the analysis of metal–PDC complexes can be achieved by extracting the complexes into organic solvents before their instrumental analysis [35,39,41,42]. In this study, ISLD-DLLME was developed for extraction of metal–PDC complexes prior to their analysis by reversed phase HPLC.

### 2.1. Separation of Metal–PDC Complexes by HPLC

The optimum mobile phase was a mixture of acetonitrile (ACN) and water at a ratio of 70/30 (v/v). Under optimum conditions, the separation of the studied metal–PDC complexes was achieved within 18 min with the order of elution of Ni(II)–PDC (11.5 min), Cr(VI)–PDC (12.9 min), Hg(II)–PDC (16.0 min), and Co(II)–PDC (17.6 min), and the excess PDC was eluted at 7.5 min. 

### 2.2. In Syringe Low Density Solvent-Dispersive Liquid Liquid Microextraction (ISLD-DLLME)

The experimental parameters that influenced the extraction efficiency were optimized, and the results of this optimization are presented in Section 2.2.1, Section 2.2.2, Section 2.2.3, Section 2.2.4 and Section 2.2.5. The average values of the peak area including the standard deviation (from triplicate experiments) were plotted either as bar or line graphs. 

#### 2.2.1. The Effect of pH

The pH of an aqueous solution has a key role in metal complex formation and extraction efficiency. The effect of pH was studied in the range of 3.0–9.0 by using a phosphate buffer. The results (Figure S1) revealed that the pH had a different effect for each metal ion. The pH slightly affected the extraction of the PDC complexes of Co^2+^ and Hg^2+^. The extraction efficiency of Ni(II)–PDC was strongly influenced by pH, as the peak area increased with the increasing of pH from 3.0 to 5.0; after that, the efficiency dramatically decreased from pH 7 to 9. While, the extraction of Cr(VI)–PDC was significantly decreased at pH levels higher than 5.0. Form the results, pH 5.0 was chosen for further studies because it provided the highest efficiency for all the studied metal ions. 

#### 2.2.2. Effect of Types and Volume of the Extraction Solvent

**Figure 1:**
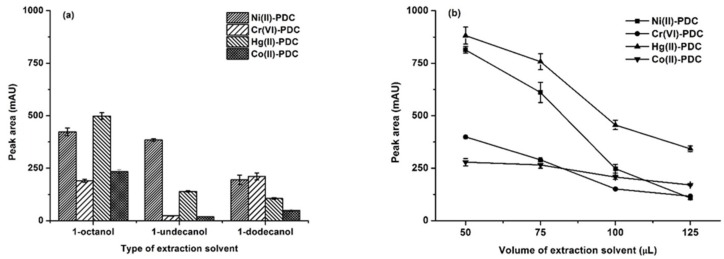
(**a**) The effect of the types of extraction solvent. Extraction conditions: 0.1 mol L^−1^ phosphate buffer at pH 5, 75 μL of extraction solvent, 300 μL of acetonitrile, and 20 s of vortex. (**b**) The effect of the extraction solvent volume. Extraction conditions: 0.1 mol L^−1^ phosphate buffer at pH 5, 50–125 μL of 1-octanol, 250 μL of methanol, and 20 s of vortex.

The type of extraction solvent is one of the most important parameters that influences extraction efficiency. The general characteristics for a good extraction solvent including (i) a high affinity to the target analytes, (ii) compatibility with HPLC separation, and (iii) a low solubility in water [20,26,43]. In addition, to be environmentally benign, the extraction solvent should have a lower density than water. The studied solvents were those that having densities lower than water, including 1-octanol, 1-undecanol, and 1-dodecanol, which their logs Kow are 3.21, 4.83 and 5.36, respectively [44]. The results (Figure 1a) showed that the most polar solvent, 1-octanol, provided the highest extraction efficiency. 

The extraction solvent volume directly affected the extraction efficiency. A sufficient extraction solvent is required for effective extraction. To investigate the effect of the extraction solvent, different volumes of 1-octanol (50, 75, 100 and 125 μL) were studied. The results shown in Figure 1b indicate that the peak area continuously decreased with the increasing volume of 1-octanol because a large volume of extraction solvent resulted in a dilution of the analytes in the extraction phase [20]. However, a volume of less than 50 μL could not be studied due to the resulting poor phase separation, so the collection of the extraction phase was difficult. Therefore, 50 μL of 1-octanol was chosen as the optimum extraction solvent volume.

#### 2.2.3. Effect of Types and Volume of the Dispersive Solvent

**Figure 2:**
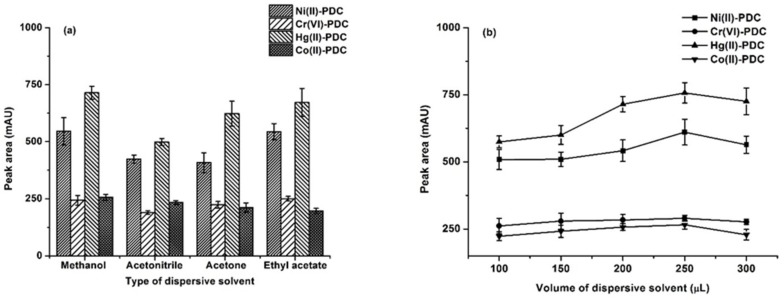
(**a**) The effect of the dispersive solvent type. Extraction conditions: 0.1 mol L^−1^ phosphate buffer at pH 5, 75 μL of 1-octanol, 300 μL of dispersive solvent, and 20 s of vortex. (**b**) The effect of the dispersive solvent volume. Extraction conditions: 0.1 mol L^−1^ phosphate buffer at pH 5, 75 μL of 1-octanol, 100–300 μL of methanol, and 20 s of vortex.

The role of the disperser solvent not only assists in the formation of tiny drops of the extraction solvent but also facilitate its dispersion. The following properties should be considered for the disperser solvent: (i) a high water solubility (ii), miscibility in the extraction solvent, and (iii) a small surface tension [28,43]. In this study, the most widely used dispersive solvents, such as methanol, acetonitrile, acetone and ethyl acetate, were investigated. The results (Figure 2a) showed that methanol gave the highest peak area. Therefore, methanol was selected as the optimal dispersive solvent.

The volumes of the dispersive solvent directly affect the cloudy phenomenon in DLLME [41,43]. The cloudy phenomenon indicates the emulsion of the fine droplets of the extraction solvent. A complete emulsion is therefore not formed if an insufficient dispersive solvent volume is used, thus leading to a decrease in the efficiency extraction. On the other hand, a large volume of dispersive solvent results in increasing volume of extraction solvent, thus decreasing the extraction efficiency. Different volumes of methanol (100, 150, 200, 250 and 300 µL) were investigated. As can be seen from the results in Figure 2b, the peak area increased and reached a maximum at 250 µL of methanol before decreasing slightly at 300 µL. Therefore, 250 µL of methanol was chosen.

#### 2.2.4. Effect of Salt Addition

**Figure 3:**
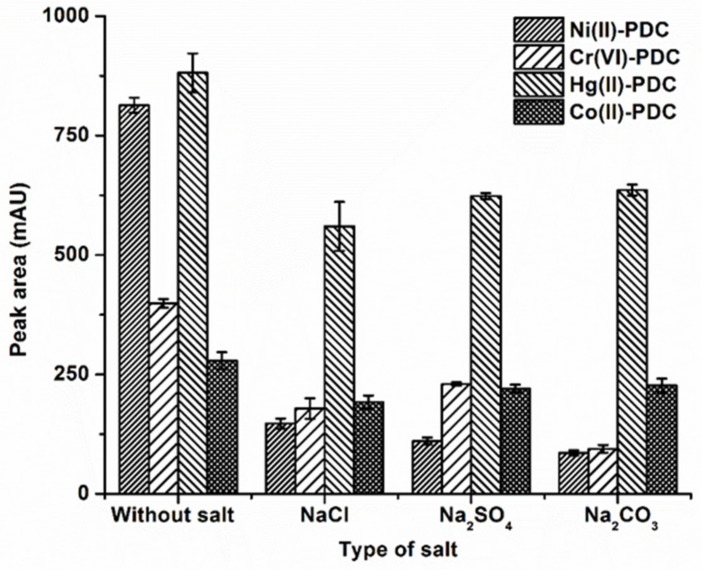
The effect of type of salt on the extraction efficiency. Extraction conditions: 0.1 mol L^−1^ phosphate buffer at pH 5, 50 μL of 1-octanol, 250 μL of methanol, 20 s of vortex, and 1 % *w*/*v* salt.

For DLLME, the addition of salt increases the extraction efficiency by decreasing the solubility of the analytes in the aqueous solution and enhancing the partitioning of analytes into the organic phase as a result of the salting out effect [31]. In this study, 1% (*w*/*v*) of various salts including NaCl, Na_2_SO_4_ and Na_2_CO_3_, were added to evaluate the effect of salt on extraction efficiency. The results are depicted in Figure 3, revealing that the peak area of all analytes decreased with the addition of salts compared to those without the salt addition. This may have been due to the fact that the addition of salts increases the ionic strength and viscosity of the aqueous phase, resulting in the decreasing of mass transfer [44]. Therefore, subsequent experiments were carried out without salt addition.

#### 2.2.5. Effect of Vortex Time

Extraction time is another important parameter. The extraction time is defined as the vortex time after the injection of a mixture of the extraction solvent and the dispersive solvent into the aqueous solution. Additionally, with the increased mixing of all reagents, a vortex can enhance the dispersion of the extraction solvent into an aqueous solution. The effect of vortex time was investigated in the range of 0–100 s. The maximum peak area was obtained at a vortex time of 20 s (Figure S2); after that, the peak area remained almost constant for the PDC complexes except for the Hg(II) PDC. Consequently, 20 s was used as the optimal extraction time.

#### 2.2.6. Analytical Features

**Figure 4:**
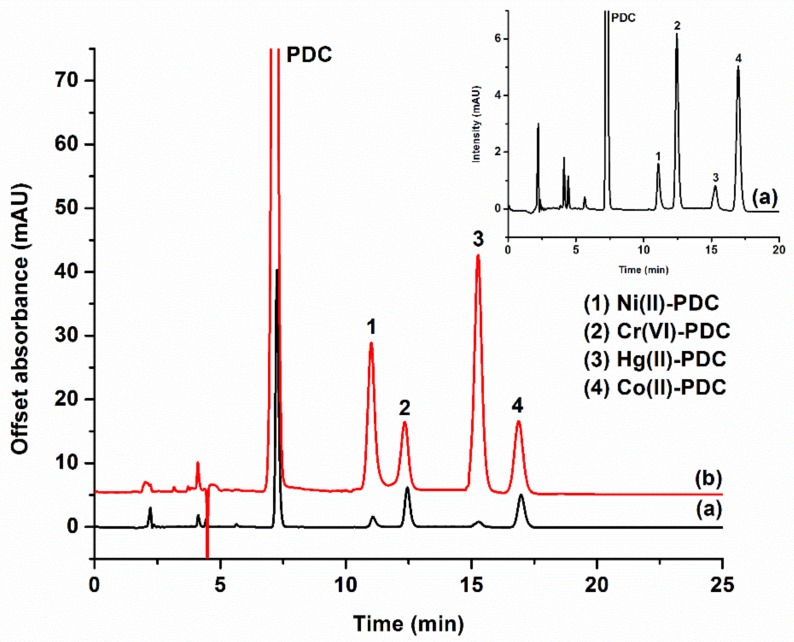
Chromatograms of the studied metals: (**a**) and (**a**) inset obtained from direct HPLC analysis PDC complexes of 0.5, 0.5, 0.5, and 1 mg L^−1^ for Ni^2+^, Co^2+^, Cr_2_O_7_^2−^, and Hg^2+^, respectively); (**b**) obtained after ISLD-DLLME (PDC complexes of 0.2, 0.01, 0.01, and 0.2 mg L^−1^ for Ni^2+^, Co^2+^, Cr_2_O_7_^2−^, and Hg^2+^, respectively).

The analytical characteristics of the proposed ISLD-DLLME were investigated by using the optimum conditions as follows: 1-octanol (50 µL) was used as the extraction solvent, and methanol (250 µL) was used as the disperser solvent—these were vortexed for 20 s. The ISLD-DLLME could significantly enhance the sensitivity for the determination of metal ions, as can be clearly seen in Figure 4. The analytical performance of ISLD-DLLME coupled to HPLC was compared to the direct HPLC analysis, and the results of this are summarized in Table 1. The linearity was deduced from the calibration curve of five different concentrations of each metal ions in the ranges of 0.050–5.0 μg L^−1^ for Ni^2+^, Cr_2_O_7_^2−^, Co^2+^, and 5.0–200 μg L^−1^ for Hg^2+^. Each metal ion exhibited a good linearity with correlation coefficients (R^2^) ranging from 0.9949 to 0.9979. The limit of detection (LOD) and the limit of quantitation (LOQ) were calculated based on the signal to noise (S/N) ratios of 3 and 10, respectively. The LODs were 0.011–0.014 μg L^−1^ for Ni^2+^, Cr_2_O_7_^2−^, and Co^2+^, and the LOD was 2.0 μg L^−1^ for Hg^2+^. The LOQs were 0.047–0.050 μg L^−1^ for Ni^2+^, Cr_2_O_7_^2−^, and Co^2+^, and the LOQ was 5.0 μg L^−1^ for Hg^2+^. Enrichment factors that were calculated from the ratio of the calibration curve slopes for the metal ions before and after their preconcentration by ISLD-DLLME were 64, 126, 204 and 230 for Ni^2+^, Cr_2_O_7_^2−^, Co^2+^, and Hg^2+^, respectively.

The proposed ISLD-DLLME was compared with the other preconcentration techniques with various chelating ligands based on DLLME, as summarized in Table 2. The ISLD-DLLME method was comparable to the other methods regarding the analytical performance in terms of sensitivity (LODs). However, ISLD-DLLME coupled to HPLC was superior to the others in respect to facility, rapidity and cost effectiveness. Furthermore, the proposed method was also preferable in regards to the eco-friendly aspect (using a less toxic solvent as the extraction solvent).

#### 2.2.7. Interference Studies

The foreign ions including cations that were capable of forming complexes with PDC were investigated as the interferences. The study was performed by individually spiking increasing amounts of foreign ions into the standard solution that contained a mixture of the PDC complexes of the studied metal ions (10 μg L^−1^ of each metal ions) before being subjected to ISLD-DLLME followed by HPLC. The results are expressed as the tolerance ratio, which is defined as the concentration ratio of the foreign ions that given the deviation of peak area of the analytes ≥5%. The results are summarized in Table S1, indicating that the studied foreign ions did not significantly affect the separation and determination of the studied metal ions.

#### 2.2.8. Analysis of Samples

**Figure 5:**
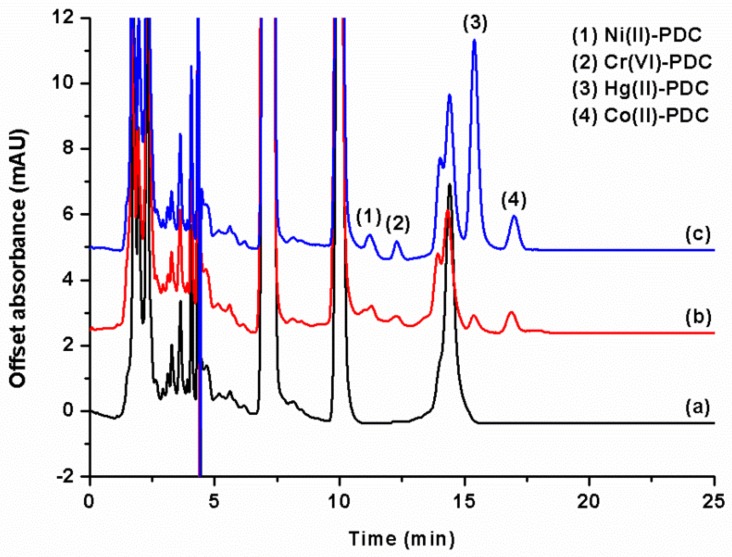
Chromatograms of *Esomus metallicus* fish. (**a**) Blank sample; (**b**) spiked sample (2.5 μg kg^−1^ for Ni^2+^, Cr_2_O_7_^2–^, and Co^2+^, and 250 μg kg^−1^ for Hg^2+^); and (**c**) spiked sample (25 μg kg^−1^ for Ni^2+^, Cr_2_O_7_^2–^, and Co^2+^, and 500 μg kg^−1^ for Hg^2+^).

To verify the applicability of the proposed method, freshwater fish, shrimp and shellfish samples were studied. To compensate the matrix effect, matrix match calibration curves with five difference concentrations of the metal ions standards were used for each sample. The studied metal ions were not detected in all the studied samples. The accuracy of the proposed method was then studied in terms of percentage recovery. The recovery was investigated by spiking known amounts of metal ions to each sample before the analysis by ISLD-DLLME and HPLC. The results are presented in Tables S2–S4 (supplementary data), and the obtained acceptable recoveries were in the range of 64.3%–114.7% (on average). Figure 5a–c depicts the typical chromatograms of the *Esomus metallicus* fish blank sample and the spiked sample; it can clearly be seen that the peaks of analytes were not interfered with by the matrix. Therefore, the proposed ISLD-DLLME combined with HPLC has the potential to detect metal ions in real samples. Moreover, there were no statistically significant differences between the results that were obtained by the developed method and ICP-OES at the 95% confidence level, as evaluated by the paired t-test. The t_critical_ value is 2.57 at degree of freedom 5 (from six samples), while the t_calculated_ values were 0.615, 0.286 and 1.40 for Ni^2+^, Cr_2_O_7_^2–^, and Co^2+^, respectively, at the spiked concentration of 2.50 µg kg^−1^ and the t_calculated_ value of 1.24 for Hg^2+^ spiked at 250 µg kg^−1^.

## 3. Experimental

### 3.1. Chemicals and Solutions

The standard solutions (1000 mg L^−1^ for AAS) of Ni^2+^, Hg^2+^, and Co^2+^ were purchased from Merck (Darmstadt, Germany), Fisher Scientific (Loughborough, UK) and Carlo Erba (Val-de-Reuil, France), respectively. The stock solution (1000 mg L^−1^) of Cr_2_O_7_^2−^ that was prepared by dissolving K_2_Cr_2_O_7_ (Ajax Finechem, Auckland, New Zealand) in a 0.01 mol L^−1^ sulfuric acid. Ammonium pyrrolidine dithiocarbamate (APDC, ≥98.0%) from Sigma-Aldrich (St. Louis, MO, USA) was used as the complexing agent.

Working standard solutions of heavy metal ions were freshly prepared via the dilution of an appropriate amount of the standard stock solutions in deionized water. 1-octanol (Panreac, Barcelona, Spain), 1-dodecanol (Sigma-Aldrich, USA) and 1-undecanol (Sigma-Aldrich, USA) were investigated as the extraction solvents. Sodium chloride (Ajax Finechem, New Zealand), sodium carbonate (Ajax Finechem, New Zealand), sodium hydroxide (Carlo Erba, France), sodium sulfate (Carlo Erba, France), acetone (Carlo Erba, France), ethyl acetate (Sigma-Aldrich, USA), 65% nitric acid (Sigma-Aldrich, USA) and 37% hydrochloric acid (QRëc, Auckland, New Zealand) were used. Methanol and ACN (HPLC grade) were purchased from Merck (Darmstadt, Germany). Buffer solutions of 0.1 mol L^−1^ phosphate (pH 3.0, 5.0, 7.0, 9.0) were used. All solutions were prepared in deionized water with the resistivity of 18.2 MΩ cm from RiOs™ Type I Simplicity 185 (Millipore, Burlington, MA, USA). Whatman no. 1 filter paper was obtained from GE Healthcare (GE Healthcare, Bangkok, Thailand), and a 0.45 μm nylon membrane filter was obtained through vertical chromatography (Vertical Chromatography, Bangkok, Thailand). All glassware in these experiments were kept in 10% (v/v) nitric acid overnight and thoroughly washed with deionized water before being used.

### 3.2. Instrumentations

The HPLC system consisted of an Agilent 1220 LC system VL, a binary pump, a manual injector with a sample loop of 10 μL, and an Agilent 1260 Infinity II Multiple Wavelength Detector (MWD, Agilent technologies, Santa Clara, CA, USA). Data acquirement and processing were performed by using OpenLAB CDS Chemstation software. A vortex mixer (50 Hz, Scientific Industries, Bohemia, NY, USA) was used to mix the solution and accelerate the phase separation. A pH meter (Model B210, ProLine, Oosterhout, The Netherlands) with a lab pH electrode was used for the pH measurements. 

A Multiwave Microwave Sample Preparation System that was equipped with an 8 SXF100 rotor, and 8 XF100 vessels (Anton Paar, Graz, Austria) were used for sample preparation.

### 3.3. In Syringe Low Density Solvent-Dispersive Liquid Liquid Microextraction (ISLD-DLLME)

**Figure 6:**
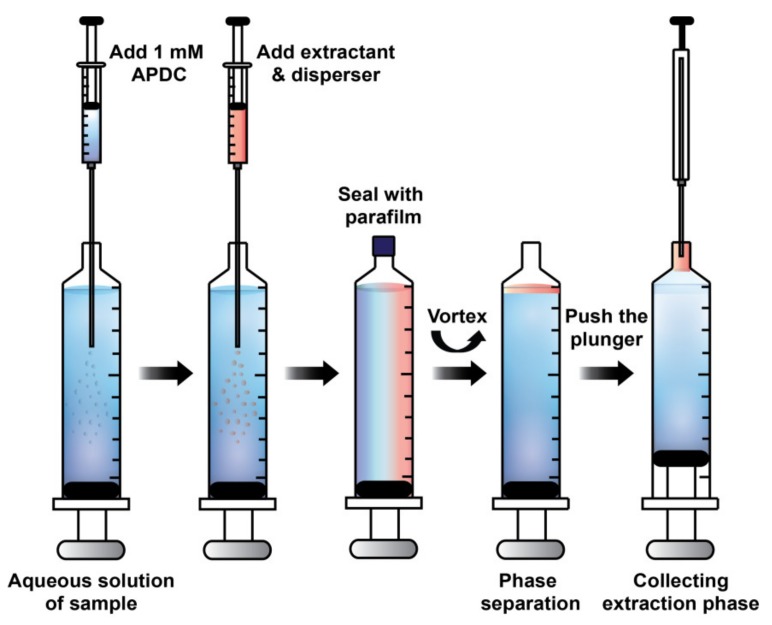
Schematic diagram illustrating ISLD-DLLME for the enrichment of the PDC complexes of Ni^2+^, Cr_2_O_7_^2−^, Co^2+^, and Hg^2+^.

An aliquot of 10.00 mL aqueous solution containing a mixture of standard metal ions and 1 mL of a 0.1 mol L^−1^ phosphate buffer was placed in a 10 mL-plastic syringe, and then 475 μL of 1 mmol L^−1^ APDC as the chelating agent was injected into the syringe. A mixture of the extraction solvent and the disperser solvent was rapidly injected, and the syringe tip was sealed with parafilm. Afterwards, the solution was vortexed at 1800 g-force, in which a cloudy solution was immediately observed. The parafilm on the tip of syringe was then removed. The extraction phase was moved into the tip of syringe by slowly pushing the plunger of syringe. The extraction phase was collected by a microsyringe and was diluted with 5 μL of methanol before being injected into HPLC for analysis. The schematic diagram for the extraction procedure of ISLD-DLLME is illustrated in Figure 6.

In order to obtain the most effective conditions, the experimental parameters that influence the extraction efficiency were optimized by investigating a single parameter at a time in the following order: pH, type and volume of extraction solvent, type and volume of disperser solvent, extraction time, and ionic strength. The extractions were carried out in a 10 mL-plastic syringe. The standard solution studied was 10 mL of aqueous solution in a mixture of 0.2, 0.01, 0.01 and 0.2 mg L^−1^ of Ni^2+^, Co^2+^, Cr_2_O_7_^2−^ and Hg^2+^, respectively, and 1 mmol L^−1^ of PDC was used throughout. All the experiments were performed in triplicate. The extraction efficiency was evaluated through a comparison of the mean peak area (n = 3) that was obtained from the chromatograms.

### 3.4. Liquid Chromatography

The chromatographic separation of the metal complexes was carried out on an ACE Excel 5 C18 AR (150 × 4.6 mm i.d., 5.0 μm) column (ACE, Aberdeen, Scotland) with isocratic elution by using a mixture of ACN and a water at ratio of 70/30 (*v*/*v*) at a flow rate of 1.0 mL min^−1^ and detection at 254 nm.

### 3.5. Sample Preparation

The studied samples were *Oreochromis niloticus*, *Esomus metallicus*, *Macrognathus siamensis*, *Saccostrea commercialis*, *Macrobrachium lanchesteri* and *Litopenaeus vannamei*. They were purchased from a local market in Khon Kaen, Thailand. The samples were prepared by taking thin slices of muscle tissue, cutting them into small pieces, and thoroughly chopping them. The samples were acid digested by using a microwave oven. A precise weight (0.2 g) of the samples was placed in a Teflon vessel. After that, 3.0 mL of 65% HNO_3_, 1.0 mL of 37% HCl, and 1.0 mL of H_2_O were added. Afterwards, the solution was filtered, and the filtrate was diluted to 25 mL with water. An aliquot (1 mL) of the diluted filtrate adjusted to pH of 5 with 5 mol L^−1^ NaOH before subjected to ISLD-DLLME (Section 3.3) and followed by HPLC analysis (Section 3.4).

## 4. Conclusions 

A simplified DLLME, namely in syringe low density solvent-dispersive liquid liquid microextraction (ISLD-DLLME) has been successfully developed for the preconcentration of the PDC complexes of Ni^2+^, Cr_2_O_7_^2−^, Co^2+^, and Hg^2+^ prior to their determination by HPLC. Besides, simplicity, rapidity of the method and environmentally friendly characteristics of the solvents used, ISLD-DLLME is also cost effective from the use of commercially available plastic syringe as the extraction device. The proposed method (ISLD-DLLME coupled with HPLC) offers sensitivity and selectivity for the simultaneous determination of the trace concentration of the studied metal ions. The applicability of the method was demonstrated for freshwater fish, shrimp and shellfish samples. Under the optimum conditions, the LODs based on the analysis of the studied samples were 0.50–100 µg kg^−1^, which are lower than DRIs and MRLs of the metal ions in fish and foods. Therefore, the proposed ISLD-DLLME method can be used as an alternative preconcentration technique for the trace analysis of metal ions. 

## Figures and Tables

**Table 1:** Analytical performance of the proposed method (in syringe low density solvent-dispersive liquid liquid microextraction (ISLD-DLLME)) for the determination of the metal ions.

Analyte	Linear Range (μg L^−1^)	Linear Equation	R^2^	LOD (μg L^−1^)	LOQ (μg L^−1^)	EF	%RSD ^*^	
							Intra-Day (n = 5)	Inter-Day (n = 3 × 5)
Ni^2+^	0.05–5 (5–500) ^a^	y = 2.681x + 4.493 (y = 0.042x − 1.654)	0.9952 (0.9951)	0.014 (1.6)	0.050 (5.7)	64	7.1 (5.1)	8.9 (7.9)
Cr_2_O_7_^2−^	0.05–5 (10–500)	y = 19.59x − 0.626 (y = 0.155x − 1.186)	0.9964 (0.9960)	0.011 (3.1)	0.049 (10)	126	5.6 (4.4)	7.0 (6.1)
Hg^2+^ Co^2+^	5–200 (500–1000) 0.05–5 (20–500)	y = 2.301x + 17.35 (y = 0.010x − 3.081) y = 34.09x + 4.304 (y = 0.167x − 2.411)	0.9979 (0.9942) 0.9945 (0.9956)	2.0 (120) 0.011 (5.8)	5.0 (460) 0.047 (18)	230 204	5.8 (2.8) 6.3 (2.7)	8.1 (4.9) 7.2 (5.2)

^a^ Value of standard without preconcentration (direct HPLC analysis). * The relative standard deviation of the LOD.

**Table 2:** Comparison of the proposed ISLD-DLLME method with other preconcentration methods for the determination of metal ions.

Method (Chelating Agent)	Sample (Metal Ions Studied)	Extraction Solvent (Dispersive Solvent)	Extractant Volume (Disperser Volume)	Extraction Time (min) ^a^	LOD (µg L^−1^)	EF	RSDs (%)	Analytical Technique	Ref.
Solvent extraction (APDC)	NR (Pb^2+^, Ni^2+^, Co^2+^, Cu^2+^, Bi^3+^, In^3+^)	Re-extraction by acetonitrile	2 mL	21	0.023–0.21	NR	≤9.2	HPLC-UV	[39]
UA-DLLME-SAP (APDC)	Water (Ni^2+^, Co^2+^, Cd^2+^, Cu^2+^, Pb^2+^)	Cyphos IL 104	10 μL	66	0.02–0.03	207–211	≤7	HPLC-UV	[23]
UA-DLLME (TOMATS)	Tea (Cd^2+^, Co^2+^, Pb^2+^)	TOMATS IL	10 μL	17	2–13	200	≤12	HPLC-UV	[45]
Automated DLLME (2-ME)	Water (Hg^2+^, MeHg^+^, EtHg^+^)	[C_6_MIM][PF_6_] (acetone)	30 μL (800 μL)	2	0.0015–0.003	41–47	≤5.1	HPLC-CVAFS	[46]
Cloud point extraction (TAN)	Water (Cr^3+^, Cr^6+^)	1.25% Triton X-114	NR	45	3.5–7.5	40–45	≤4.7	HPLC-UV	[47]
ISLD-DLLME (APDC)	Fish, shrimp, shellfish (Ni^2+^, Cr_2_O_7_^2−^, Co^2+^, Hg^2+^)	1-octanol (Methanol)	50 μL (250 μL)	1	0.01–2	64–230	≤8.9	HPLC-UV	This work

^a^ Extraction time: total time in preconcentration procedures. NR: not reported. TOMATS IL: Trioctylmethylammonium thiosalicylate ionic liquid. 2-ME: 2-Mercaptoethanol. TAN: 1-(2-thiazolylazo)-2-naphthol. UA-DLLME-SAP: ultrasound-assisted dispersive liquid–liquid microextraction based on the solidification of the aqueous phase.

## Supplementary Materials

The following are available online, Figure S1: The effect of the pH on the extraction efficiency, Figure S2: The effect of the extraction time on the extraction efficiency, Table: Condition wet digestion for microwave, Table S1: Effect of interference ions on the detection of metal ions, Table S2: The determination of metal ions and recovery in *Oreochromis niloticus fish* and *Esomus metallicus fish*, Table S3: The determination of metal ions and recovery in *Macrognathus siamensis fish* and *Macrobrachium lanchesteri shimp*, Table S4: The determination of metal ions and recovery in *Litopenaeus vannamei shrimp* and *Saccostrea commercialis shellfish*, Table S5: Results of the accuracy of the extraction procedure.
