# Supplementary material for: A Simple in Syringe Low Density Solvent-Dispersive Liquid Liquid Microextraction for Enrichment of Some Metal Ions Prior to Their Determination by High Performance Liquid Chromatography in Food Samples
Source: Molecules. 2020 Jan 28;25(3):552. doi: 10.3390/molecules25030552 (PMC7037012; doi:10.3390/molecules25030552)
Supplement: Supplementary file 1 [file molecules-25-00552-s001.pdf]

Supporting Information

A Simple In Syringe Low Density Solvent-Dispersive Liquid Liquid Microextraction for Enrichment of Some Metal Ions Prior to Their Determination by High Performance Liquid Chromatography in Food Samples

Melasinee Laosuwan[a], Siriboon Mukdasai[a], Supalax Srijaranai[a]*

[a]*Materials Chemistry Research Center, Department of Chemistry and Center of Excellence for Innovation in Chemistry, Faculty of Science, Khon Kaen University, Khon Kaen 40002, Thailand*

* Corresponding author:
Tel.: +66 43 202222 to 41 ext. 12243; fax: +66 43 202373
E–mail address: supalax@kku.ac.th (S. Srijaranai)

1. The effect of pH

The pH of aqueous solution has a key role in the metal complex formation and extraction efficiency. The effect of pH was studied in the range of 3.0-9.0 using phosphate buffer. The results (**Fig. S1**) reveal that the pH had different effect for each metal ion. The pH affected slightly on the extraction of PDC complexes of Co^{2+} and Hg^{2+}. The extraction efficiency of Ni(II)-PDC was strongly influenced by pH which peak area increased with the increasing of pH from 3.0 to 5.0, after that the efficiency dramatically decreased from pH 7 to pH 9. While, the extraction of Cr(VI)-PDC was significantly decreased at pHs higher than 5.0. Form the results, pH 5.0 was chosen for further studies as it provided the highest efficiency for all the studied metal ions.

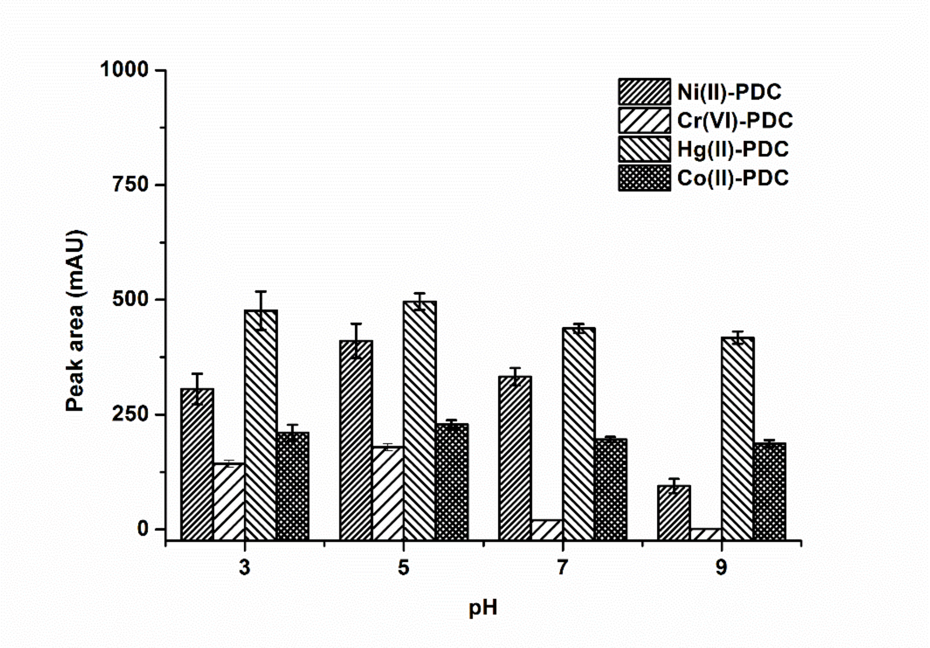

Fig. S1 The effect of the pH on the extraction efficiency. Extraction conditions: 0.1 mol L^{-1} phosphate buffer pH (3-9), 1-octanol 75 µL, acetonitrile 300 µL and vortex 20 s.

2. Effect of vortex time

Extraction time is another important parameter. The extraction time is defined as the vortex time after the injection of a mixture of extraction solvent and dispersive solvent into the aqueous solution. Besides, increasing well mixing of all reagents, vortex can enhance dispersion of the extraction solvent into the aqueous solution. The effect of vortex time was investigated in the range of 0-100 s. The maximum peak area was obtained at vortex time of 20 s (**Fig. S2**), after that the peak area remained almost constant for PDC complexes except for Hg(II) PDC. Consequently, 20 s was used as the optimal extraction time.

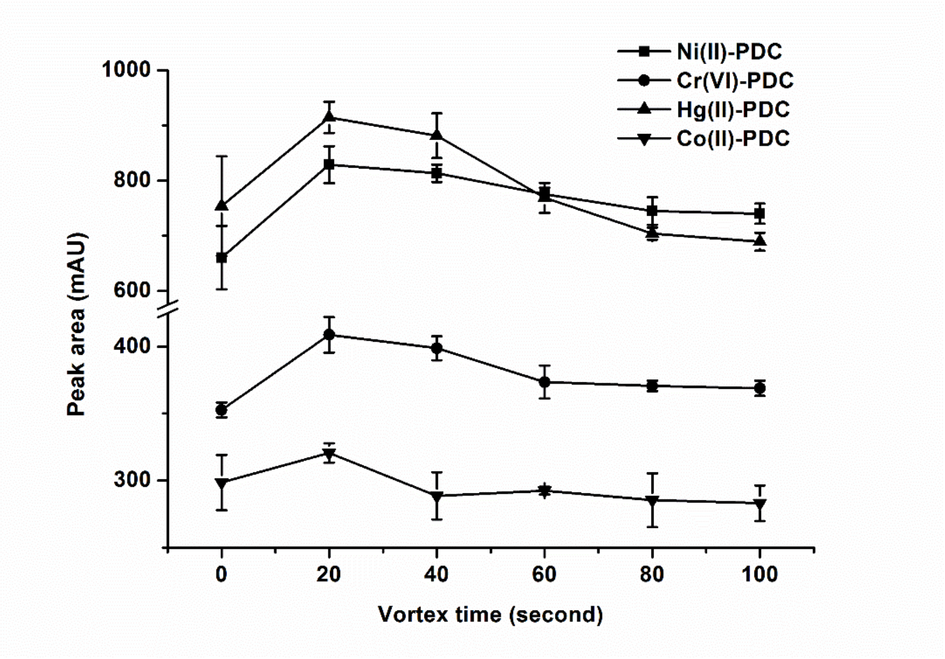

Fig. S2 The effect of the extraction time on the extraction efficiency. Extraction conditions: 0.1 mol L^{-1} phosphate buffer pH 5, 1-octanol 50 μL, methanol 250 μL and vortex time 0-100 s.

3. Sample preparation

Table Condition wet digestion for microwave.

Step	Power (W)	Ramp (min)	Hold time (min)	Fan
1	300	05:00	10:00	1
2	400	05:00	15:00	1
3	0	0	05:00	3

4. Interference studies

The foreign ions including cations capable to form complexes with PDC were investigated as the interferences. The study was performed by individually spiking increasing amounts of foreign ions into the standard solution containing a mixture of the PDC complexes of the studied metal ions (10 μg L^{-1} of each metal ions) before subjected to ISLD-DLLME followed by HPLC. The results are expressed as the tolerance ratio which defined as the concentration ratio of the foreign ions that given the deviation of peak area of the analytes ≥5%. The results are summarized in **Table S1**, indicating that the studied foreign ions were not significantly affect the separation and determination of the studied metal ions.

Table S1 Effect of interference ions on the detection of metal ions

Interference ion	Tolerance ratio
As^{3+}	1250
Cd^{2+}	100
Pb^{2+}	100
Mn^{2+}	1500
Cr^{3+}	1000
Zn^{2+}	1000
Fe^{2+}	50
Fe^{3+}	1000
Ca^{2+}	5000
Mg^{2+}	2500

Table S2 The determination of metal ions and recovery in *Oreochromis niloticus fish* and *Esomus metallicus fish* (n=3).

**Analyte**	**Spiked ($\mu g\ kg^{-1}$)**	*Oreochromis niloticus fish*			*Esomus metallicus fish*		
		**Found ($\mu g\ kg^{-1}$)**	**Recovery (%)**	**RSD (%)**	**Found ($\mu g\ kg^{-1}$)**	**Recovery (%)**	**RSD (%)**
**Ni^{2+}**	0.0	ND	-	-	ND	-	-
	2.5	1.785	71.4	8.9	2.002	80.1	7.7
	25	20.95	83.8	7.2	19.60	78.4	6.9
	50	39.18	78.4	5.6	46.01	92.0	6.1
**$Cr_2O_7^{2-}$**	0.0	ND	-	-	ND	-	-
	2.5	2.352	94.1	7.9	2.625	104.9	8.7
	25	23.03	92.1	6.4	23.91	95.6	5.5
	50	34.05	68.0	4.1	37.05	74.1	5.1
**Hg^{2+}**	0.0	ND	-	-	ND	-	-
	250	200.2	80.1	6.8	228.6	91.4	7.1
	500	432.3	86.5	4.4	401.1	80.2	3.9
	2500	2096	83.8	3.1	2261	90.4	2.8
**Co^{2+}**	0.0	ND	-	-	ND	-	-
	2.5	2.030	81.2	8.5	1.901	76.0	7.4
	25	22.33	89.3	5.3	18.63	74.4	5.5
	50	40.21	80.4	2.8	46.15	92.3	3.2

Table S3 The determination of metal ions and recovery in *Macrognathus siamensis fish* and *Macrobrachium lanchesteri shimp* (n=3).

**Analyte**	**Spiked ($\mu g\ kg^{-1}$)**	*Macrognathus siamensis fish*			*Macrobrachium lanchesteri shimp*		
		**Found ($\mu g\ kg^{-1}$)**	**Recovery (%)**	**RSD (%)**	**Found ($\mu g\ kg^{-1}$)**	**Recovery (%)**	**RSD (%)**
**Ni^{2+}**	0.0	ND	-	-	ND	-	-
	2.5	1.678	67.1	8.2	2.268	90.7	8.5
	25	20.95	83.8	7.8	17.62	70.4	7.5
	50	39.30	78.6	7.3	37.25	74.5	6.4
**$Cr_2O_7^{2-}$**	0.0	ND	-	-	ND	-	-
	2.5	2.350	93.8	7.7	2.183	87.3	8.9
	25	21.43	85.7	6.4	20.05	80.2	6.6
	50	38.21	76.4	4.2	40.66	81.3	4.5
**Hg^{2+}**	0.0	ND	-	-	ND	-	-
	250	176.1	70.4	7.3	160.8	64.3	7.8
	500	357.0	71.4	5.9	330.4	66.1	4.3
	2500	2254	90.2	2.2	2320	92.8	2.1
**Co^{2+}**	0.0	ND	-	-	ND	-	-
	2.5	2.400	95.5	8.1	2.209	88.4	8.8
	25	27.53	110.1	6.5	20.77	83.1	6.7
	50	54.01	108.0	4.4	43.14	86.3	4.9

Table S4 The determination of metal ions and recovery in *Litopenaeus vannamei shrimp* and *Saccostrea commercialis shellfish* (n=3).

**Analyte**	**Spiked ($\mu g\ kg^{-1}$)**	*Litopenaeus vannamei shrimp*			*Saccostrea commercialis shellfish*		
		**Found ($\mu g\ kg^{-1}$)**	**Recovery (%)**	**RSD (%)**	**Found ($\mu g\ kg^{-1}$)**	**Recovery (%)**	**RSD (%)**
**Ni^{2+}**	0.0	ND	-	-	ND	-	-
	2.5	1.928	77.1	7.6	2.016	80.6	8.4
	25	21.55	86.2	6.3	21.62	86.4	6.2
	50	38.35	76.7	6.7	39.40	78.8	6.5
**$Cr_2O_7^{2-}$**	0.0	ND	-	-	ND	-	-
	2.5	2.868	114.7	8.8	2.538	101.5	4.4
	25	21.10	84.4	7.2	21.28	85.1	5.1
	50	44.65	89.3	5.1	41.40	82.8	5.8
**Hg^{2+}**	0.0	ND	-	-	ND	-	-
	250	194.5	77.8	7.1	173.5	69.4	7.9
	500	481.0	96.2	3.2	387.1	77.4	7.1
	2500	2368	94.7	2.9	1980	79.2	4.7
**Co^{2+}**	0.0	ND	-	-	ND	-	-
	2.5	2.145	85.8	8.6	2.257	90.3	3.9
	25	18.02	72.1	7.7	17.93	71.7	5.2
	50	37.64	75.3	6.5	36.10	72.2	6.1

Table S5 Results of the accuracy of the extraction procedure.

Metal ions	Concentration (μg L^{-1})	%Recovery
Ni^{2+}	0.05	98.5 ± 0.2
	0.1	86.1 ± 0.4
	1	94.7 ± 0.7
	3	100.8 ± 0.9
	5	99.5 ± 0.7
$Cr_2O_7^{2-}$	0.05	91.0 ± 0.3
	0.1	93.3 ± 0.7
	1	86.9 ± 1.2
	3	83.6 ± 3.3
	5	92.6 ± 4.7
Hg^{2+}	5	97.2 ± 1.3
	25	96.1 ± 3.6
	50	104.4 ± 2.7
	100	106.6 ± 6.4
	200	109.9 ± 5.2
Co^{2+}	0.05	102.7 ± 0.5
	0.1	96.9 ± 1.9
	1	98.8 ± 2.4
	3	92.9 ± 5.9
	5	97.2 ± 6.6
